# Targeting the Warburg Effect in Anaplastic Thyroid Carcinoma: Metabolic Vulnerabilities and Therapeutic Opportunities

**DOI:** 10.3390/ijms27125472

**Published:** 2026-06-17

**Authors:** Olga-Maria Iova, Gheorghe-Eduard Marin, Vlad Răzniceanu, Ștefania-Maria Mocrei-Rebrean, Sebastian Romeo Pintilie, Romana T. Netea-Maier, Ioana Berindan-Neagoe

**Affiliations:** 1 Faculty of Medicine, University of Medicine and Pharmacy “Iuliu Hațieganu”, 400023 Cluj-Napoca, Romania; iova.olga.maria@elearn.umfcluj.ro (O.-M.I.);; 2 Department of Genomics, MEDFUTURE Institute for Biomedical Research, University of Medicine and Pharmacy “Iuliu Hațieganu”, 400012 Cluj-Napoca, Romania; 3 Department of Internal Medicine, Division of Endocrinology, Radboud University Medical Center, 6525 GA Nijmegen, The Netherlands; 4 Doctoral School, University of Medicine and Pharmacy “Iuliu Hațieganu”, 400012 Cluj-Napoca, Romania; 5 Academy of Medical Sciences, 030167 Bucharest, Romania

**Keywords:** Warburg effect, anaplastic thyroid cancer, poorly differentiated thyroid cancer, lactate metabolism, aerobic glycolysis

## Abstract

Anaplastic thyroid carcinoma (ATC) represents the most aggressive thyroid malignancy, characterized by rapid progression, therapeutic resistance, and poor prognosis. Conventional treatments remain largely ineffective, highlighting the need for novel therapies. Metabolic reprogramming, particularly the Warburg effect (WE), has emerged as a promising area of investigation. This review synthesizes current evidence on the role of WE in ATC and PDTC, integrating data from molecular profiling, preclinical studies, and emerging therapeutic strategies. Oncogenic alterations frequently observed in ATC, including mutations in BRAF, RAS, TP53, and activation of PI3K/AKT/mTOR and HIF-1α signaling, converge to promote glycolytic reprogramming. This metabolic shift supports tumor proliferation, immune evasion, and metastasis through increased glucose uptake, lactate production, and microenvironmental remodeling. Key metabolic nodes, including glucose transporters, hexokinase, and monocarboxylate transporters, are regarded as promising targets. Preclinical studies suggest that pharmacological inhibition of these pathways reduces tumor growth, enhances radiosensitivity, and improves response to targeted therapies. Future efforts should focus on combination therapies, biomarker-driven patient stratification, and the development of targeted delivery systems to overcome toxicity and resistance. A deeper understanding of tumor metabolic heterogeneity will be essential for translating these approaches into clinical practice.

## 1. Introduction

Thyroid cancer is the most frequent endocrine malignancy, accounting for over 90% of all diagnosed endocrine cancers and almost 50,000 deaths per year, a figure which is expected to increase in the following decades [1]. As the incidence rises, the need for new therapeutic methods is also escalating, as traditional therapies are often futile or insufficient for certain patient groups. Current strategies aim to directly disrupt metabolic pathways that are overexpressed in cancer cells compared to healthy cells. As cancer metabolism differs profoundly from that of normal cells, certain hyperregulated pathways, such as glycolysis, become prime targets for oncological therapies. This hyperregulation of the glycolysis pathway is known as the Warburg effect (WE). In this review, we consider specific WE inhibitors as possible therapeutic strategies in aggressive thyroid cancers.

Most thyroid cancers follow a relatively mild clinical course, with a high survival rate. Specific subtypes, however, behave aggressively and demand more intensive, targeted treatment. The rarest and most aggressive form of thyroid malignancy is Anaplastic Thyroid Cancer (ATC), which, although originating from follicular cells, lacks any resemblance to normal cytoarchitecture or function, being entirely or partially composed of undifferentiated cells [2,3,4]. Despite representing less than 5% of all thyroid malignancies, ATC is responsible for a significant percentage of thyroid carcinoma deaths (14–39%) [5]. An entity that bridges the gap between well-differentiated thyroid carcinoma and ATC has also been described: poorly differentiated thyroid carcinoma (PDTC), a rare disease with an estimated incidence of less than 10% among all thyroid neoplasms [6]. PDTC can itself progress to ATC [7]. Both are highly aggressive and lethal diseases, representing a disproportionately high percentage of thyroid cancer deaths. For ATC, overall survival was 35–59% at 1 year and 50% or less at 3 years, with a median survival time from 3 months to 1.31 years [8,9]. The low survival rates of these diseases contrast with often inefficient multimodal management options, treatment being often palliative in nature, as classical therapies such as radioactive iodine therapy, chemotherapy, and radiotherapy are rendered useless [10,11,12].

Advances in our understanding of cancer pathophysiology and oncogenic progression have highlighted several key metabolic pathways as potential therapeutic targets. Among these is glycolysis, a tightly regulated metabolic process that becomes abnormal in many cancers, including aggressive thyroid neoplasms, leading to the phenomenon known as the Warburg effect (WE) or aerobic glycolysis [13]. The WE describes the preferential use of glycolysis over mitochondrial respiration, even in the presence of sufficient oxygen, leading to increased lactate levels [14]. Inhibiting the WE could offer a therapeutic approach to disrupt the cancer cells’ reliance on aerobic glycolysis for energy and biosynthetic building blocks. By targeting critical enzymes and transporters that maintain glycolytic flux and lactate export, these strategies aim to reestablish oxidative metabolism and inhibit tumor growth [15]. Overall, treating both cancer types remains challenging because of their rapid evolution and limited effective treatment options, making the development of new therapies an ongoing need. Targeting specific pathways, such as glycolysis in the context of WE, either alone or in combination, could offer promising results in treating these aggressive thyroid cancers.

This article reviews the WE inhibitors as treatment options for aggressive thyroid malignancies, identifying which metabolic components are most critical for ATC progression. By comparing the efficacy of various metabolic targets and their preclinical evidence, this review suggests possible alternatives for existing therapies and outlines essential future directions for the field.

## 2. Pathogenesis and Mutational Profiling in ATC

In dedifferentiated thyroid cancers, the WE extends beyond energy metabolism, acting as a central regulator of tumor plasticity through lactate-driven signaling, epigenetic modulation, and microenvironmental remodeling. Moreover, multiple convergent pathways specific to aggressive thyroid cancers result in increased energetic needs and an intensification of the WE. While these mutational burdens establish the genetic makeup of malignancy, their primary impact lies in the profound reprogramming of cellular function. Specifically, these oncogenic drivers converge to shift the cell’s bioenergetic priorities, transitioning from efficient mitochondrial respiration to the rapid, glycolytic metabolism, in the form of WE. In this review, we critically evaluate specific oncogenic pathways responsible for the malignant transformation of ATC and their effect in hyperregulating WE.

ATC represents the most dedifferentiated and lethal forms of thyroid malignancies. These pathologies exist along a biological continuum of tumor progression: while well-differentiated neoplasms, such as papillary (PTC) and follicular (FTC) thyroid cancers, typically harbor a single driver mutation, ATC is characterized by a high mutational burden that drives their aggressive phenotypes, with a median of 1 mutation in PTC, 2 in PDTC, and 6 in ATC [16]. Although ATC arises from the same follicular cells as differentiated thyroid cancers, its pathogenesis diverges through rapid accumulation of secondary mutations, leading to loss of cellular differentiation [17]. Despite being an aggressive thyroid malignancy, PDTC generally exhibits a less fulminant clinical course compared with ATC, with improved survival outcomes and a greater likelihood of response to conventional therapies. This difference is largely attributed to its intermediate degree of differentiation, as PDTC retains partial thyroid-specific functional and metabolic characteristics that are progressively lost in ATC. Additionally, PDTC remains less studied than ATC, and the lack of experimental studies further complicates the possibility to compare them.

In mutational profiling, certain key driver mutations have been identified as influencing the pathogenesis of thyroid malignancies, with specific mutations carrying prognostic and therapeutic value. While the mutational profiles of PDTC and ATC share several key drivers, their genomic landscapes differ in distinct ways that influence both disease progression and therapeutic response.

Mutations in the BRAF and RAS genes, both part of the RAS-RAF-MAPK pathway, which is known to promote cell proliferation, dedifferentiation, and survival in ATC [18], are mutually exclusive [16] and are common to PDTC and ATC, albeit with different frequencies. BRAFV600E occurs less frequently in PDTC (33%) than in ATC (45%). Mutations in BRAF have been shown to directly influence the MAPK/ERK pathway, leading to increased proliferation, migration, and invasion [19,20,21]. Wen et al. showed that the BRAFV600E/p-ERK/p-DRP1(Ser616) pathway increased WE by upregulating glycolytic enzymes and suppressing mitochondrial respiration in PTC [22], while Nagarajah showed that BRAFV600E-positive thyroid tumors exhibit increased glucose uptake, a characteristic of WE tumors [23].

RAS mutations have the same frequency in both ATC and PDTC (24–27%) [16,19,20] and are known to promote the switch from oxidative phosphorylation to glycolysis by upregulating glycolytic enzymes and increasing glucose uptake. Emerging evidence suggests that RAS mutations also engage the PI3K–AKT–mTOR pathway, thereby enhancing glucose transporter expression, glycolytic flux, protein synthesis, and anabolic metabolism, which further reinforces the WE. Although data in ATC and PDTC remain limited, this may partly explain the link between RAS alterations and metabolic reprogramming [24,25,26].

PPARγ is a nuclear hormone receptor with mainly metabolic and possible tumor-suppressive roles and is weakly expressed in the thyroid tissue. The PAX8/PPARγ rearrangement on the other hand is a thyroid oncoprotein which most commonly fuels the progression of differentiated thyroid carcinomas, by possibly contributing to metabolic reprogramming in thyroid tumorigenesis through altered regulation of genes involved in glucose metabolism. By disrupting normal PPARγ signaling, this fusion may influence cellular differentiation status and metabolic homeostasis, potentially shifting tumor cells toward a more glycolytic phenotype [27]. While PPARγ is usually measurable in ATC cell lines or tumor samples, especially as nuclear staining, PAX8/PPARγ is usually not detectable, indicating a possible different tumorigenic pathway. Moreover, agonists of PPARγ, such as Efatutazone, have been used as treatment in ATC patients [28].

TERT promoter mutations, clonal in both PDTC and ATC, are the most common mutation in PDTC (40%), and in ATC (65–73%) [16,20]. In both cancers, TERT promoter alterations are associated with BRAF and RAS mutations, and although they do not directly regulate glycolysis, they cooperate to create the aggressive, glucose-dependent phenotype specific to ATC/PDTC [16,29,30].

Mutations in tumor suppressor genes such as p53, which are linked to increased aerobic glycolysis metabolism in multiple cancer types [31,32,33], are found in both cancer types but are less frequent in PDTC than in ATC [16,34]. p53 is a tumor suppressor gene which normally suppresses glycolysis and promotes mitochondrial respiration. However, when mutated, aside from reversing these effects, it also marks the progression from differentiated cancer types to aggressive invasive forms, such as ATC [35,36].

Other mutations, such as ATM, a gene involved in cell-cycle checkpoint control and DNA repair, are found equally often in the two cancers [16]. When mutated, ATM leads to oxidative stress which disrupts mitochondrial function, increasing Hypoxia Inducible Factor 1α (HIF-1α), thus leading to overexpression of glycolytic enzymes and establishing WE [37,38,39]. HIF-1α activation in ATC represents a central factor of WE. It promotes metabolic reprogramming by upregulating glucose transporters (e.g., GLUT1) and glycolytic enzymes (e.g., HK2, PFK, and LDHA), while simultaneously suppressing mitochondrial oxidative phosphorylation through induction of pyruvate dehydrogenase kinase (PDK1). Beyond hypoxia and ATM-induced oxidative stress, HIF-1α is also increased because of oncogenic MAPK and PI3K/AKT/mTOR pathways, highlighting its role as a central convergence node linking genetic alterations to metabolism adaptations in aggressive thyroid cancers [5,40,41].

Members of the PI3K/AKT/mTOR pathway, such as PTEN, are less frequently mutated in PDTC than in ATC [16,19,20]. This pathway has been shown to promote WE in both cancer cells and inflammatory macrophages [42], aside from sustaining cancer growth, invasion and metastasis in numerous cancers including ATC/PDTC [43,44].

Current data suggests that the intricate interplay between oncogenic driver mutations, aberrant signaling pathways, and metabolic reprogramming must be considered when designing future combination therapies. However, there is currently no consensus on how to stratify ATC/PDTC-specific mutations by therapeutic potential, as only a limited subset is targeted, either directly or in combination. Most notably, the BRAF^V600^ inhibitor dabrafenib, particularly combined with the MEK inhibitor trametinib, is currently used in clinical practice with satisfactory results [45]. AKT inhibitor capivasertib has shown promising results in certain solid tumors such as breast cancer [46]. In contrast, other mutations have either shown intrinsic resistance to targeted approaches or lack clinically approved therapies altogether. Further research is therefore essential to determine the clinical potential of these mutations, develop safe and effective therapies, and optimize combination strategies for clinical use.

Collectively, these findings suggest that the WE in ATC and PDTC is not driven by a uniform metabolic program, but rather by partially distinct oncogenic routes, principally MAPK, PI3K/AKT/mTOR, p53, and hypoxia/HIF-1α mediated pathways, which converge to produce a shared glycolytic phenotype in the form of WE.

Differences in mutational profiling seen in differentiated thyroid cancers and ATC/PDTC are listed in Table 1.

## 3. Warburg Effect in Dedifferentiated Thyroid Cancers

### 3.1. Core Metabolic Hallmarks of the WE

Cancer metabolism differs profoundly from that of normal cells, reflecting its capacity for rapid, uncontrolled proliferation and metastatic spread. Key properties, such as sustaining proliferative signaling, evading growth suppressors, inducing angiogenesis, and activating invasion and metastasis, allow cancer cells not only to develop, grow, and obtain resources but also to evade immune surveillance and resist cell death, as first described by Hanahan [54,55,56]. Key oncogenic genes and pathways influence these cancer properties, as previously discussed.

One hallmark of cancer, namely modified energy metabolism, describes alterations in energy-producing pathways that are rewired to support the growing energy and nutrient demands of the cancer cell [57]. These alterations target glucose metabolism, including both glycolysis and oxidative phosphorylation, as well as lipid and glutamine metabolism [58]. Recent years have seen a rise in interest in targeting these pathways to cut off the cancer cell’s energy supply, making it vulnerable to therapy.

A prominent therapeutic strategy involves inhibiting glycolysis to exploit the metabolic vulnerabilities of malignant cells. This approach targets a shift first identified in the 1920s by Otto Warburg, who observed that cancer cells convert glucose into lactate at rates significantly higher than those of healthy tissues. Known as WE, this phenomenon describes the neoplastic preference for glycolysis as a major ATP source over oxidative phosphorylation, even when oxygen is abundant. Because this shift occurs despite sufficient oxygen levels, the WE is frequently termed aerobic glycolysis [59].

Although less efficient at ATP production than the Krebs cycle, the WE is the essential part of cancer cell metabolism [60]. It has been suggested that, in the primary environment of early cancers and in the tumor center, due to a general lack of oxygen caused by insufficient blood supply, aerobic glycolysis might provide a survival advantage [61]. 

Additionally, it has been postulated and later confirmed by theoretical and bacterial methods that, although glycolysis produces less ATP per unit of glucose than oxidative phosphorylation, its catalytic rate is 10–100 times faster. This allows for a significantly higher temporal yield of ATP, compensating for lower efficiency with sheer metabolic speed [14,62,63].

Crucially, we do not suggest a complete loss of mitochondrial function in thyroid cancer cells. While some cells depend on aerobic glycolysis, others maintain oxidative phosphorylation as their main ATP source. This is demonstrated by the ‘Reverse WE,’ a symbiotic relationship in which primarily cancer-associated fibroblasts (CAFs) undergo glycolysis and secrete lactate, which nearby cancer cells then use to convert it back into pyruvate to fuel the Krebs cycle. This metabolic relationship allows the tumor to optimize energy efficiency across its diverse microenvironment [64,65]. Moreover, this symbiotic relationship can also occur between cancer cells, as hypoxic cells located inside tumors release lactate, which is then used by oxygenated tumor cells in the Krebs cycle [66].

### 3.2. Functional Contributions to Tumor Progression

Research suggests that WE may influence the survival of cancer in both the initial stages of development as well as during proliferation [15]. WE has been described in a multitude of cancers, including gastrointestinal, lung, breast, head and neck, and thyroid cancers, highlighting the importance of understanding this key metabolic pathway and targeting it therapeutically [67,68]. The reprogramming of glucose metabolism could also influence several cancer hallmarks, providing the energy needed for proliferation [69], apoptotic resistance [70], angiogenesis [71,72], and metastasis [61,73], making WE a central pivot in cancer pathophysiology. The pathway of aerobic glycolysis partially mirrors that of normal glycolysis, though it is dysregulated, with certain key enzymes and transporters altered [15]. In normal cells, glucose enters the cell through specialized transmembrane proteins called glucose transporter (GLUT), which regulate intracellular glucose concentration [74]. Once inside the cytoplasm, glucose is transformed through a series of catalytic steps into the final product, pyruvate, along with two ATP molecules [75]. The pyruvate can then enter the Krebs cycle or be metabolized into other products, such as lactate. These reactions are influenced by the cell’s oxygenation state and its metabolic needs and are highly regulated [76].

In cancer cells, glycolytic enzymes are markedly upregulated, driving a metabolic shift toward lactate production to satisfy the immense bioenergetic and biosynthetic demands of malignancy. This metabolic reprogramming is orchestrated by key upstream regulators, as previously discussed in Chapter 2, including the PI3K/Akt/mTOR and RAS signaling pathways, as well as HIF-1α [77]. These pathways collectively enhance glycolytic flux by upregulating glucose transporters and rate-limiting enzymes, further accelerating glycolysis, sustaining high metabolic rates even under normoxic conditions [78,79]. In contrast, tumor suppressors like p53 oppose the WE by promoting oxidative metabolism and repressing GLUT1. A mutated p53 on the other hand, removes this brake on glycolysis and promotes GLUT1 translocation to the plasma membrane [80].

**Figure 1:**
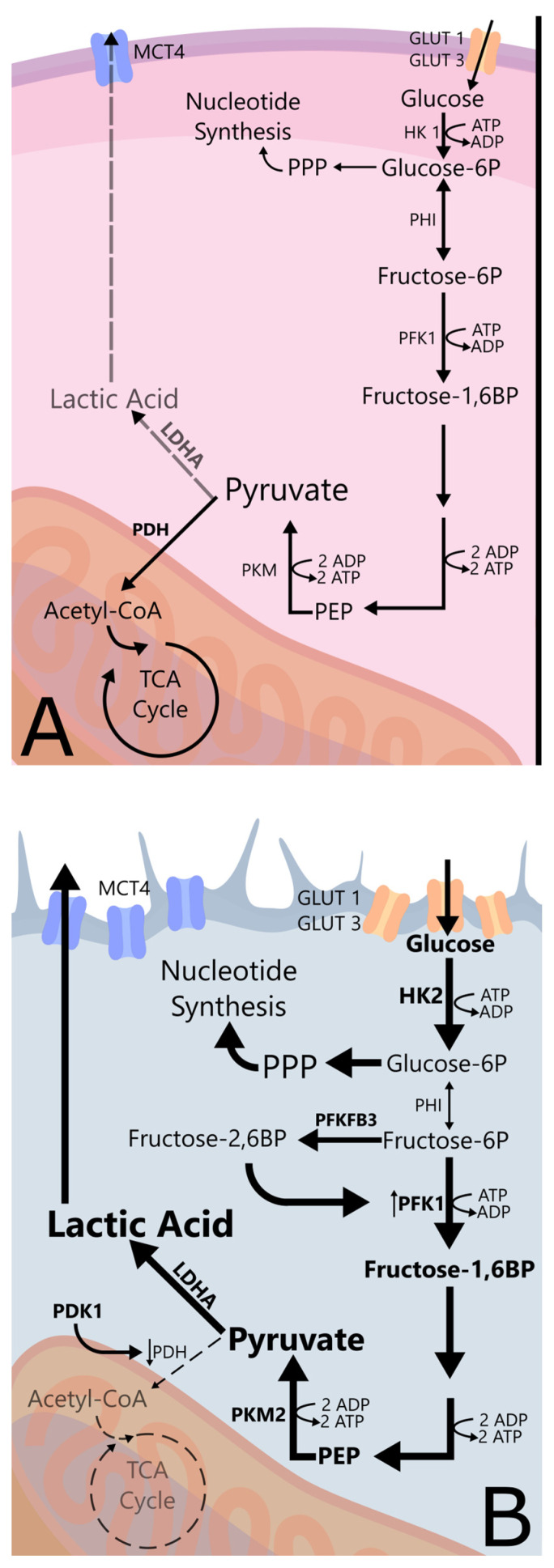
Comparative representation of glycolysis and lactate metabolism in a normal cell (**A**) vs. a cancerous cell displaying WE (**B**). Thin arrows: primary physiological reaction; enlarged arrows: pathologically hyperregulated reaction; dotted arrow: down-regulated reactions. The image presents the most important changes in enzyme expression and the preferred pathways employed by normal versus cancerous cells. ADP, Adenosine Diphosphate; ATP, Adenosine Triphosphate; Fructose-1,6BP, Fructose-1,6-Bisphosphate; Fructose-2,6BP, Fructose-2,6-Bisphosphate; Fructose-6P, Fructose-6-Phosphate; Glucose-6P, Glucose-6-Phosphate; GLUT, glucose transporter; HK, hexokinase; LDHA, Lactate Dehydrogenase A; MCT4, Monocarboxylate Transporter 4; PDH, Pyruvate Dehydrogenase; PDK1, Pyruvate Dehydrogenase Kinase 1; PEP, Phosphoenolpyruvate; PFK1, Phosphofructokinase 1; PFKFB3, 6-phosphofructo-2-kinase/fructose-2,6-biphosphatase 3; PHI, Phosphohexose Isomerase; PKM, Pyruvate Kinase M; PKM2, Pyruvate Kinase M2; PPP, Pentose Phosphate Pathway; TCA Cycle, Tricarboxylic acid cycle.

The main differences between physiological and aerobic glycolysis are shown in Figure 1.

### 3.3. WE in ATC

Focusing on the specific metabolic alterations characteristic of ATC, it is essential to consider the unique intersection of thyroid-specific metabolism and oncogenic signaling. Therefore, we must address the effects of thyroid metabolism and the hormonal regulatory loops that contribute to the development of the metabolic reprogramming characteristic of these malignancies. Key properties of the normal thyroid cell, such as iodine uptake, TSH regulation, and hormone production, are dysregulated or absent during oncogenesis [13,81,82]. TSH is the primary hormone acting on the thyroid. Under normal conditions, it stimulates thyroid cells by binding to the TSH receptor (TSHR) on their surface. Stimulating the G-protein-coupled receptor increases iodine absorption, thyroid hormone production, and thyroid follicle size [83]. TSH also increases glucose uptake and stimulates intracellular glucose metabolism through cAMP [35]. Thyroid hormone production, a process tightly coordinated by TSH, is a multi-step process involving the synthesis of thyroglobulin, iodine absorption, iodination of thyroglobulin, storage and release of T3 and T4, and, finally, deiodination. All these processes are highly regulated, with multiple feedback loops acting on the hypothalamic-pituitary-thyroid axis [84].

In thyroid cancers, these pathways become dysregulated, especially in ATC. Although TSH can act as a growth factor in the early stages of oncogenesis [85], in later stages of dedifferentiated cancer development, the sensitivity to TSH/TSHR is lost, making cancer cells no longer responsive to changes in TSH levels and rendering this therapeutic target ineffective [86]. Iodine uptake within cells is also absent in ATC, making radioactive iodine therapy also inefficient [87]. Another notable difference between ATC and differentiated thyroid cancers is the overexpression of deiodinase 2, an enzyme present in thyroid cells that converts circulating T4 into T3. Recent data suggest that deiodinase is directly involved in ATC proliferation by providing high levels of T3, which is necessary for cancer growth, and that blocking it causes cells to enter growth arrest and senescence, while also reducing their invasion potential [88,89,90]. 

Another important pathway in normal thyroid cells is the pentose phosphate pathway (PPP), which converts ribose-5-phosphate into nucleotides and generates NADPH in the process [91], a critical cofactor for hormone synthesis [92]. However, during oncogenesis, especially in ATC, PPP becomes hyperregulated to meet the high metabolic needs of cancer cells, including nucleotide and antioxidant production [81]. In this regard, Liu et al. showed that inhibiting the PPP in an ATC cell line led to cell apoptosis due to increased oxidative stress [93]. PPP is also closely linked to WE, as the high uptake of glucose leads to the redirection of glucose-6-phosphate towards PPP [94].

As previously mentioned, ATC is characterized by aggressive behavior, early metastasis, and rapid local growth, and current data suggest that extracellular lactate, a byproduct of the WE, may contribute to and sustain these mechanisms. Lactate has emerged as a possible mediator of several cancer properties, by directly influencing key cellular components of the tumor microenvironment (TME), such as cancer-associated fibroblasts (CAFs) and immune cells, or indirectly, through mechanisms including histone lactylation, upregulation of glycolytic enzymes, stabilization of HIF-1α, and reduction in microenvironmental pH. Lactate thus facilitates immune suppression, maintenance of the Warburg phenotype, angiogenesis, invasion, and metastasis, by closely collaborating with CAFs [95].

**Figure 2:**
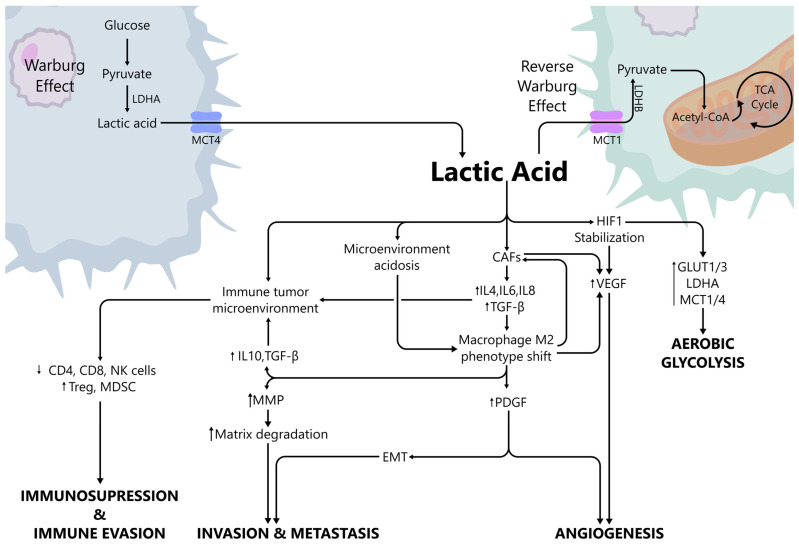
The Metabolic Crosstalk Between ATC Cells and the Tumor Microenvironment. The progression of Anaplastic Thyroid Cancer (ATC) is characterized by a sophisticated metabolic interplay between malignant cells and the tumor microenvironment (TME), primarily mediated by lactate acting as an extracellular signaling molecule. Lactate is generated via the Warburg Effect (WE) within ATC cells or other tumor-associated components, such as fibroblasts. It is subsequently exported into the extracellular space through the monocarboxylate transporter MCT4. Once in the TME, lactate follows divergent pathways: it may persist within the acidified micromedium or be internalized by oxidative cancer cells via MCT1. In the latter, lactate is converted back into pyruvate to fuel mitochondrial respiration, a metabolic process termed the Reverse Warburg Effect. Beyond its role as a metabolic fuel, extracellular lactate also regulates tumor dynamics, as presented above. CAFs, Cancer associated fibroblasts; CD4, Cluster of differentiation 4, Helper T cells; CD8, Cluster of differentiation 8, Cytotoxic T cells; EMT, Epithelial–mesenchymal transition; GLUT1/3, Glucose transporters 1 and 3; HIF1, Hypoxia-inducible factor 1α; IL, Interleukin; LDHA, Lactate dehydrogenase A; LDHB, Lactate dehydrogenase B; MCT1, Monocarboxylate transporter 1; MCT4, Monocarboxylate transporter 4; MDSC, Myeloid-derived suppressor cell; MMP, Matrix metalloproteinase; NK, Natural killer cells; PDGF, Platelet-derived growth factor; TCA Cycle, Tricarboxylic acid cycle; TGF-β, Transforming growth factor β; Treg, Regulatory T cell; VEGF, Vascular endothelial growth factor. Up arrows: increased level/expression, down arrows: decreases levels/expression.

When stimulated by high lactate concentrations and the concomitant decrease in pH, CAFs modulate the activity of macrophages, shifting their phenotype to the immunosuppressive M2 state, and other immune populations, decreasing levels of CD4 and CD8 T cells and NK cells, leading to a decrease in the immune response, while simultaneously increasing immunosuppressive Treg cells and myeloid-derived suppressor cells [96,97]. This paracrine signaling cascade facilitates a shift toward immunosuppression and immune evasion, while simultaneously driving invasion and metastasis. M2 Macrophages also play a pivotal role in the TME, by influencing immune processes, angiogenesis, matrix degradation, and cancer cell migration [98]. While the relationship between lactate and the TME is exceedingly complex, this model highlights the pivotal role of the WE in orchestrating tumor progression. Finally, lactate is a key player in the reverse WE, as discussed above [99,100]. Possible pathological links are shown in Figure 2, which analyzes the relationships among ATC cells, the tumor microenvironment, and processes such as invasion and metastasis.

In the context of cancer metabolism, it is noteworthy to mention that although WE is essential for the progression of ATC, many other aberrant metabolic pathways are also involved. The interactions and intersections between glycolytic metabolism and amino acid, nucleotide, or lipid metabolism in cancer cells, though of utmost importance, are beyond the scope of this paper and have been discussed extensively elsewhere [13,81].

### 3.4. Current Evidence and Knowledge Gaps for Warburg Effect Inhibition in PDTC

Compared with ATC, evidence regarding the WE and its therapeutic targeting in PDTC remains limited. Although PDTC exhibits metabolic reprogramming and increased glycolytic activity, these alterations appear less pronounced and more heterogeneous than those observed in ATC. Most available studies have focused on ATC-derived models, while PDTC specific investigations are rare, making it difficult to determine whether the metabolic vulnerabilities identified in ATC are equally relevant in PDTC [7,101].

Current knowledge regarding WE inhibition in PDTC is largely derived from studies that include PDTC cell lines alongside ATC models. However, few studies have specifically evaluated glycolysis-targeting agents in PDTC as a distinct biological entity. Consequently, the relative contribution of glycolysis to tumor growth, progression, and therapeutic resistance in PDTC remains incompletely understood. Furthermore, it is unclear whether the metabolic vulnerabilities identified in ATC, including dependence on lactate export, pyruvate metabolism, or HIF-1α signaling, are equally relevant in PDTC [7,13,101]. A more cautious approach should be employed when comparing the two entities.

## 4. Metabolic Targets in WE

While WE is often characterized as a singular metabolic hallmark, its execution relies on a complex network of specific enzymatic drivers. In aggressive malignancies such as ATC, identifying these molecular targets is paramount, as they represent the vulnerabilities that can be exploited for targeted therapeutic intervention. Here, we examine the most important and studied enzymes involved in sustaining WE in cancer cells.

Targeting enzymes involved in glycolysis may be therapeutically beneficial, especially when combined with other classical treatment approaches. While preclinical evaluations of several glycolytic inhibitors have demonstrated varying degrees of efficacy, a detailed analysis of their therapeutic potential and current limitations is provided in Chapter 5. We first examine the enzymes involved in glycolysis and lactate metabolism and their dysregulation in the context of WE, followed by a characterization of the metabolic signatures unique to ATC, The enzymatic drivers of the WE, along with their roles in sustaining malignant metabolism, are summarized in Table 2.

### 4.1. Transporters

#### 4.1.1. GLUT

As a polar molecule, glucose requires the assistance of a transporter to enter the cell. Specialized glucose transporters, called GLUTs, are intramembrane proteins that facilitate the entry of glucose from the extracellular medium into the cytoplasm [119]. As key factors in controlling the amount of glucose that undergoes glycolysis and thus directly affecting the magnitude of WE, they are critical in cell proliferation and are evident in pathological processes [15,120].

Studies have shown that cancer cells upregulate GLUT protein expression to meet their high metabolic and energetic demands. Especially, GLUT1 and GLUT3 have been found predominantly in several cancers and are also involved in chemoresistance and metastasis [15,121].

Nahm et al. analyzed 556 thyroid cancer samples to compare differences in glycolytic metabolism. In thyroid cancers, GLUT expression was elevated compared with benign nodules or normal tissue, with the highest GLUT1 levels in ATC compared with PTC, suggesting it may be a marker of aggressiveness and dedifferentiation [104]. Kim et al. determined that GLUT1 is expressed preferentially in ATC and PDTC, with 85.7% and 11.1% of biopsies positive, respectively, compared to differentiated thyroid malignancies, which were 0%. This contrasts with the results of other studies, which showed that PTC and FTC also stained positively for GLUT-1. The differences may arise from differences in staining technique, as Kim et al. imposed stricter positivity criteria [122]. Finally, Ciampi et al. showed that both GLUT-1 and GLUT-3 are overexpressed in aggressive dedifferentiated thyroid cancers [123], possibly indicating a higher metabolic rate [64].

High levels of GLUT1 were also associated with high metastatic capacity seen in aggressive cancers, possibly explaining the early metastasis observed in ATC [64,124].

Considering these results, GLUT could be an attractive therapeutic target, given its direct role in influencing WE and its potential for combination with radiotherapy and/or chemotherapy.

#### 4.1.2. MCT

Monocarboxylate transporters (MCTs) are intramembranous proteins that facilitate bidirectional transport of lactate into and out of the cell [64]. Two isoforms are particularly relevant to cancer metabolism: MCT1, which mediates intracellular lactate influx, and MCT4, which mediates lactate efflux [125]. Efficient lactate efflux is critical for sustaining glycolytic flux; by preventing intracellular acidification and regenerating the NAD^+^ required for upstream reactions, this export ensures that cancer cells can meet their high energetic and biosynthetic demands [120].

High expressions and activity of MCT proteins have been correlated with aggressiveness and tumor progression [116]. Nahm et al. found that higher expression of MCT4 in PDTC led to a worse prognosis and overall survival [104].

Johnson et al. found higher MCT1 expression in ATC cells, and thus higher lactate uptake, compared to normal thyroid tissue and PTC, possibly indicating a higher incidence of the reverse WE in these cells [126].

Preclinical data suggests that MCT1 inhibitors could also be used in combination with other therapeutic lines, especially immunotherapy. Studies on animal models have shown how dual targeting increases T cell penetration in various solid tumors, leading to a better response and possibly reversing the immunosuppressive tumor microenvornment [127,128].

### 4.2. Glycolysis Enzymes

#### 4.2.1. Hexokinase

Hexokinase (HK) catalyzes the first reaction of glycolysis, converting glucose to glucose-6-phosphate. Two isoforms are essential in cancer cell metabolism: HK type 1, a ubiquitous enzyme present in normal cells, and HK type 2, which is usually overexpressed in many types of cancers [61,81,129], and whose levels have been correlated with a poorer prognosis. As a rate-limiting enzyme, HK2 could represent a compelling therapeutic target.

Considering thyroid cancer metabolism, Rijksen et al. found no difference in HK2 expression between ATC and well-differentiated medullary thyroid carcinoma [130]. In contrast, Verhagen et al. found that HK2 levels were higher in dedifferentiated thyroid cancer patients than in PTC patients [131], a result similar to that reported by Nahm et al. [104]. Aggarwal et al. cultured ATC cell lines in high or low glucose concentrations, showing that HK2 levels are greater in the high concentration [132].

HK2 inhibitors could therefore represent a rationale for further therapeutic exploration, given their pro-apoptotic properties, especially when combined with other treatments, such as diet or radiotherapy, although their clinical translation remains to be validated [133].

#### 4.2.2. PFKFB3

6-phosphofructo-2-kinase/fructose-2,6-biphosphatase 3 (PFKFB3), although not directly involved in glycolysis, has emerged in recent years as a possible key regulator in WE. It acts by converting Fructose-6-Phosphate into Fructose-2,6-Biphosphate (F-2,6BP), which, in turn, is a potent activator of Phosphofructokinase-1 (PFK1). PFK1 in turn converts Fructose-6-Phosphate into Fructose-1,6-Biphosphate, a rate limiting step in the process of glycolysis. High levels of PFKFB3 indirectly increase the rate of glycolysis through F-2,6BP, and thus affect the magnitude of WE [134]. Regarding cancer progression, RAS mutations have been shown to influence PFKFB3 levels [110]. An ubiquitous enzyme, its levels have been found to be increased in various types of cancer, including ATC, where it possibly influences cell proliferation and migration [110,135].

#### 4.2.3. PKM2

Pyruvate kinase M (PKM) is the last enzyme in glycolysis, catalyzing the rate-limiting conversion of phosphoenolpyruvate to pyruvate. Increased levels were detected across multiple cancer types and were associated with high glycolytic activity [15]. Cells modulate PKM activity through the alternative splicing of the PKM gene, yielding the distinct isoforms PKM1 and PKM2. While PKM1 is typically expressed in high-energy-demand tissues, the PKM2 variant is markedly upregulated under oncogenic conditions. As a central driver of WE, PKM2 simultaneously increases glycolytic flux and anabolic activity [136,137].

Apart from its enzymatic role, PKM2 can also bind to HIF-1α, increasing its activity and resulting in the overexpression of key WE enzymes, [15,120,138], as well as act directly as a transcription factor influencing gene transcription, cell migration, adhesion, and metastatic progression [120,139]. HIF-1α can also upregulate PKM2 expression, thus forming a feed-forward loop [140].

Studies have also correlated the level of expression of PKM2 with a worse prognosis, decreased overall survival, and decreased disease-free survival, including for thyroid cancer patients. However, whether this upregulation actively drives disease progression or serves primarily as a prognostic biomarker remains to be fully elucidated [141]. Xu et al. showed that alternative splicing of the PMK gene through RBX1, a component of the ubiquitin ligase, in ATC patients increases PKM2 expression, thereby positively impacting the WE magnitude and decreasing overall survival [137]. Kachel et al. found that PKM2 levels were highest in malignant thyroid tissue, with the highest levels in dedifferentiated thyroid cancer samples [142]. Regarding HIF-1α, previous studies reported high nuclear staining in ATC, supporting the relationship between PKM2 and HIF-1α [143]. Similar to the other WE proteins mentioned above, inhibition of PKM2 increases cancer cells’ sensitivity to various chemotherapies and radiotherapy in preclinical settings, making it a promising therapeutic target [144,145].

### 4.3. Lactate Metabolism

#### 4.3.1. LDH

Lactate dehydrogenase (LDH) is a bidirectional enzyme that converts pyruvate into lactate. Its role is essential for aerobic glycolysis, as this conversion is required to sustain glycolytic reactions. Lactate dehydrogenase A (LDHA) and lactate dehydrogenase B (LDHB) catalyze the same reversible interconversion of pyruvate and lactate. However, due to their distinct kinetic properties, LDHA preferentially drives the conversion of pyruvate to lactate, whereas LDHB more efficiently promotes the oxidation of lactate to pyruvate [146].

LDHA and lactate levels have been correlated with tumor progression, aggressiveness, and chemoresistance [121,147]. LDHA levels were elevated in both thyroid cancer cultures and patient samples, highlighting a potential therapeutic target [148,149]. Kachel et al. found that LDHA levels were highest in malignant thyroid tissue, with the highest levels observed in dedifferentiated thyroid cancer samples [142], which supports the potential use as targets in aggressive thyroid cancers like ATC and PDTC.

#### 4.3.2. PDK1

Although not directly involved in lactate metabolism, PDK1 shifts pyruvate metabolism toward lactate conversion by inhibiting the pyruvate dehydrogenase complex, the enzyme that converts pyruvate to Acetyl-CoA, thereby inhibiting mitochondrial respiration. This is especially important in highly hypoxic tumors, as hypoxia is a trigger for mitochondrial death and ROS secretion. By inhibiting mitochondrial activity, PDK1 prevents the secretion of ROS, thus protecting the tumor from their toxic effects [150].

PDK1 has been found to be up-regulated in aggressive tumors via HIF-1α, MYC, and EGFR, thus linking specific oncogenes and transcription factors to WE [151].

Although direct studies of PDK1 expression in ATC are limited, other studies indicate high expression in PTC, where it is associated with rapid growth and metastasis, as well as in highly hypoxic tumors [152,153].

### 4.4. Transcription Factors

#### HIF-1α

HIF-1α has both direct and indirect effects on WE. As a transcription factor, it regulates the expression of key WE enzymes, while also inhibiting the conversion of pyruvate to Acetil-CoA and impairing mitochondrial function, leading to mitophagy [154,155]. Its high expression has been linked to aggressive tumors, rapid growth, and early metastasis. It is normally absent from normal thyroid tissue, making it an attractive target for therapies [156]. Induced by both hypoxia and PI3K/AKT and MAPK signaling pathways, which are usually associated with thyroid carcinomas, HIF-1α is an important metabolic regulator in thyroid malignancies. The highest nuclear staining for HIF-1α has been observed in ATC cells, suggesting a potential therapeutic target [143].

Table 3 presents landmark studies investigating the expression levels of key glycolytic enzymes in ATC and PDTC, while also summarizing the information presented above.

## 5. Studies Using Warburg Inhibitors and Targeted Pathway Nodes in ATC and Comparison Between Them

As described above, WE is a complex phenomenon with profound implications for cancer development and progression, especially in ATC/PDTC, with a multitude of enzymatic steps and regulators. These regulatory pathways create multiple targets for pharmacological disruption of cancer metabolism. Targeting the WE primarily relies on small-molecule inhibitors that block critical glycolytic enzymes and transporters. In parallel, genetic interventions such as RNA interference and CRISPR-based knockouts are widely used in preclinical research to validate these molecular targets and reproduce the associated metabolic effects [157]. Here, we discuss the most important studies using WE inhibitors in ATC/PDTC. So far, all studies have been preclinical, with minor exceptions, in vitro or in vivo, and no studies have examined more than one inhibitor; no comparisons have been made. Criteria such as Half maximal inhibitory concentration (IC_50_) could be used to compare them, as well as their effects on normal thyroid cell lines. Further research is needed to fully assess the clinical potential of these inhibitors, including their potency against cancer cells and, preferably, their minimal impact on normal thyroid tissue.

A variety of small-molecule inhibitors have been developed to target key nodes of cancer glycolysis and lactate production/export.

A prime example of this is glucose transport inhibitors, which limit substrate uptake, thereby starving cancer cells. Inhibitors of GLUT1, such as BAY-876 and STF-31, have shown significant reductions in colony number in the ATC cell line 8505C, as well as synergistic interactions with lenvatinib [158].

Shikonin, a natural naphthoquinone, inhibited PKM2 activity, glycolysis, and lactate production, as well as GLUT1 expression, in a dose-dependent manner in an in vitro model of ATC [159]. Furthermore, apigenin, a plant-derived flavonoid, was administered to reduce GLUT1 and GLUT3 expression in SW1736 ATC cells [160].

Inhibition of PFKFB3 (e.g., by 3PO, PFK158) reduces fructose-2,6-bisphosphate levels, thereby suppressing PFK1 activity and slowing glycolytic flux; this has been observed to impair tumor cell proliferation and reduce vessel sprouting in preclinical models [161,162].

According to a recent multiomics and bioinformatics analysis, Aurora-A is a protein kinase that phosphorylates PFKFB3, promoting glycolysis and metabolic reprogramming. Alisertib’s inhibition of the Aurora A kinase has been suggested to halt tumor progression in ATC cell lines (8305C, 8505C, CAL62) and in vivo by impairing PFKFB3-mediated glycolysis and enhancing sensitivity to Sorafenib [163].

KAN0438757, a derivative of 3-(3-pyridinyl) -1-(4-pyridinyl) -2-propen-1-one, has been studied as a direct inhibitor of the key glycolytic enzyme PFKFB3 in ATC cell lines KHM-5M and CAL62. Furthermore, in mice injected with CAL62 cells, it suppressed tumor growth and decreased lactate production [135].

Lactate export may be blocked with AZD3965 (MCT1-selective; active in MCT4-low tumors and now in Phase I) or dual MCT1/MCT4 strategies (e.g., syrosingopine), which disrupt lactate export, acidify the cytosol, and, especially with metformin, drive synthetic lethality via NAD^+^ depletion [164,165,166]. In ATC specifically, Zhao et al. showed that pharmacologic inhibition of lactate shuttles with acriflavine, syrosingopine, and AZD3965 in 8505C, JL30, and TCO1 cells under low-glucose conditions significantly reduced lactate secretion, proliferation, and glycolytic capacity. In that study, combining AZD3965 with acriflavine further suppressed cell growth, supporting the concept that simultaneous disruption of glycolysis and lactate export is particularly effective in highly glycolytic ATC cells [167].

Additionally, regulatory-axis agents such as HIF-1α antagonists (e.g., acriflavine) and pathway modulators (e.g., mTOR inhibitors) have been shown to downregulate HIF-1-dependent glycolytic genes in preclinical models [41,91]. A study reported promising results for acriflavine as an anti-HIF-1α agent that modulates MYC signaling in CAL62 and 8305C cell lines. Acriflavine suppressed ATC cell growth and proliferation at low micromolar doses and reduced tumor volume in vivo without affecting animal weight [168]. Given the mechanistic link between MYC overexpression and the WE [169], acriflavine may be a suitable candidate for WE inhibition research in ATC. A study by Aprile et al. used small interfering RNA to block HIF-1α and found that this decreased glycolytic gene expression, glucose uptake, lactate efflux, and cell viability in a BRAF-mutant ATC cell line. Using the BRAF inhibitors, which act by blocking the BRAF-MEK-ERK signaling and thus decreasing HIF-1α, glucose uptake in cells and lactate release were diminished. Moreover, they showed that stabilizing HIF-1α’s activity leads to increased resistance to B-raf inhibitors [170].

In an ATC xenograft model, LDHA inhibition led to growth inhibition, but without regression or cure. In preclinical models, complete tumor regression was achieved only when combined with radiotherapy [171]. This suggests that LDHA may sensitize tumor cells to radiotherapy, providing a rationale investigation as an adjuvant in classical therapies.

2-Deoxyglucose (2-DG) serves as a glycolytic inhibitor by competing for glucose transport and HK phosphorylation. Experimental evidence indicates that combining 2-DG with radiotherapy yields synergistic antitumor effects. In xenograft mice, 2-DG significantly decreased tumor signal intensity by HP-MRI. The data provided by Sandulache et al. is encouraging for in vivo ATC experiments using alternative antiglycolytic agents with superior pharmacokinetics [172]. Additionally, 2-DG in 8305C/HA-RBX1 and CAL62/shRBX1 cells blocked glycolytic flux in a dose-dependent manner [137].

A recent study showed that inhibiting autophagy in ATC cells shifts mitochondrial respiration toward glycolysis. 2-DG treatment resulted in greater reductions in ATC cell viability when combined with autophagy impairment and a reduction in oxidative phosphorylation via the carnitine palmitoyltransferase 1 inhibitor etomoxir. The same study also suggests that the BRAF inhibitor vemurafenib also inhibits glycolysis in ATC cells, though the mechanism was not detailed [173].

In a similar mechanism of action, Zhao et al. found that 3-BP, an HK2 inhibitor, reduced glycolysis and dramatically slowed the growth of ATC cell line 8505C. Moreover, combined ketogenic diet and 3-BP treatment improved disease outcomes in ATC mice [174].

Mannose is an isomer of glucose and inhibits glycolysis by being phosphorylated by HK into mannose-6-phosphate. PLX4032 potentiates the effect of mannose in ATC cells 8305C and 8505C by inhibiting phosphomanose isomerase activity and thus promoting mannose-6-phosphate accumulation. The co-administration of PLX4032 and mannose resulted in superior tumor size reduction in cell-derived xenograft tumors in vivo compared to PLX4032 [175].

MG132, a proteasome inhibitor, indirectly modulates glycolysis by stabilizing SMAR1 through RBX1 inhibition in ATC cells (8305C and CAL62). Xu et al. found that PKM2 and RBX1 protein levels were significantly enhanced in ATC tissues. RBX1 regulates PKM alternative splicing to promote PKM2-mediated WE in ATC cells. RBX1 knockdown with subtype-specific shRNA in CAL62 cells resulted in a reduction in PKM2 expression in CAL62 cells, lower Glucose-6-phosphate levels, lactate generation, and ATP production [137].

As mentioned previously, PDK1 is a major enzyme of glucose oxidation which has been shown to be upregulated in experimental models under the influence of oncogenic tyrosine kinases, promoting the WE [176,177,178]. Various PDK1 inhibitors, of which the most widely studied is dichloroacetate (DCA), have been successfully used to reduce cancer cell proliferation and metastasis [176,177,179]. DCA acts by binding the pyruvate-binding domain of PDK1 and is the only PDK1 inhibitor with promising clinical efficacy in glioblastoma, although with varying results and toxic side effects, especially neurotoxicity [180,181,182].

Few studies investigate PDK modulation in thyroid cancer. One study suggested that PDK4 expression and CAL62 cell proliferation and glucose consumption could be significantly limited by silencing circCCDC66 [183]. Another study showed that artemisinin induces reversible time-dependent changes in PDK1 levels in ATC model CAL62 cells [184]. It could be that the scarcity of research on PDK inhibitors in thyroid cancer partially owes itself to the issue of establishing which PDK isoform is overexpressed across different tumor types [151] and the fact that achieving therapeutic DCA concentrations involves liver and neurotoxic effects [185].

Finally, the Ph-responsive and nucleus-targeting platinum nanocluster (Pt@TAT/sPEG) may suppress the WE in the ATC cell line 8505C by limiting LDH activity, reducing lactate and ATP production, and increasing ROS levels, while its presence in an orthotopic xenograft murine model reduces tumor growth [186]. Furthermore, diclofenac has been found to inhibit LDHA activity, as well as GLUT1 and MCT4 transporters, and to impair glucose uptake and lactate excretion in 8505C cells [170]. The dose- and time-dependent inhibition of LDH via a doxycycline-inducible shRNA system reduced ATCHth83 cell proliferation and increased radiation sensitivity, leading to tumor cell death [171]. PLX4032/vemurafenib (a BRAF inhibitor which reduces pErk) and Trametinib (a MEK inhibitor) have been demonstrated to inhibit glucose uptake, lactate production, and to suppress glycolysis-related gene expression in ATC cell line 8505C [170]. Silencing the tyrosine kinase receptor RON with a specific siRNA in 8305C cells impaired glycolysis by downregulating HK2, PKM2, and MAPK/CREB signaling, thereby elevating the chemotherapy response [187].

The mechanisms of action, experimental models, and dosage data for the WE inhibitors reported in the literature regarding ATC/PDTC have been summarized in Table 4.

## 6. Discussion: Challenges, Limitations, and Future Perspectives in Targeting Cancer Metabolism in ATC

### 6.1. Current Limitations

Over the past several decades, cancer therapeutics have evolved rapidly, leading to the development of novel targeted interventions that have significantly altered the oncology treatment paradigm. Despite staggering progress, certain types of cancer remain indifferent to modern therapies, underscoring the need for innovative treatments.

Dedifferentiated cancers remain the most difficult to treat tumors, partly due to a lack of targeted therapy and partly due to their aggressive behavior. ATC is no exceptions, as traditional therapies have shown limited efficacy, and alternative therapies have yet to be proven effective in large-scale studies. The distinct metabolic reprogramming observed in ATC poses significant challenges, as cancer cells can rapidly switch among multiple metabolic pathways to survive under various therapeutic pressures and bypass single-pathway inhibitors. Therefore, metabolic plasticity of cancer cells allows them to switch to alternative metabolic pathways, relying on oxidative phosphorylation, lipid metabolism, glutamine metabolism, or other carbon sources, limiting therapeutic efficacy in the case of isolated therapies [189]. Targeting multiple pathways, such as glycolysis, lipid metabolism, one-carbon metabolism, or glycogen use, simultaneously can be not only complicated and cost-prohibitive but also dangerous and toxic to patients.

Considering PDTC, another limiting factor is the lack of studies investigating it as a sole entity. The available experimental evidence is heavily skewed toward ATC models, with most included studies relying on ATC cell lines such as 8505C, CAL62, and 8305C, whereas PDTC-specific data are sparse and largely limited to isolated models (e.g., BCPAP). This imbalance restricts the generalizability of the findings to PDTC. Extrapolation of ATC-derived results to PDTC should be made with caution. While WE is present in both malignancies, it is generally less pronounced in PDTC, where tumors retain more metabolic flexibility and partial oxidative metabolism. In contrast, ATC exhibits a stronger and more consistent glycolytic dependency, reflecting its more complete dedifferentiation and higher metabolic reprogramming. While substantial preclinical evidence supports targeting the WE in ATC, evidence in PDTC remains limited. Future studies should specifically investigate whether the metabolic vulnerabilities identified in ATC are also present in PDTC before therapeutic strategies are generalized across both entities.

None of the WE inhibitors previously discussed are currently used in clinical settings as a standard of care. However, there have been small-sample trials or phase 1 clinical trials (MCT inhibitors AZD3965 and CYT-0851) investigating the clinical effects and safety of WE inhibitors, as well as some ongoing phase 2 clinical trials (PDK1 inhibitor DCA in patients with glioblastoma). Other substances, such as 3-BP, which, although entered promising clinical trials, were discontinued due to toxicity. This is further complicated by the lack of comparative studies between inhibitors, as none examine more than one WE inhibitor simultaneously. Moreover, even studies investigating inhibitors of the same enzyme fail at times to determine the IC_50_ values specific to each cell line or standardize quantification methods of effectiveness. Therefore, it is difficult to assess the effectiveness of blocking one enzyme at the expense of others, or whether a combination approach, by simultaneously inhibiting different targets, could prove effective and feasible.

### 6.2. Effectiveness of WE Inhibitors and Their Limitations

We can further speculate on which inhibitors are most likely to emerge as attractive therapeutic candidates by comparing their in vitro efficacy, clinical development status (where available), and expected toxicity profiles. Although many compounds show strong anti-tumor effects in vitro, their translational potential depends primarily on whether efficacy is sustained in animal models and whether systemic metabolic inhibition can be tolerated in human patients. Among the agents mentioned, a small subset has progressed beyond in vitro to in vivo and clinical settings.

Regarding the toxicity of in vitro inhibitors, we must recognize their impact on essential pathways in healthy cells. While some side effects can be managed with supportive therapy, others can be life-threatening.

Some WE inhibitors which were used in clinical settings (such as DCA, 3-BP, 2DG) led to adverse effects ranging from mild to life threatening, thus limiting their clinical translation. Normal tissue with high metabolic needs, such as the nervous system, liver, kidney, or heart, is the first affected, leading to neurotoxicity (DCA), hepatotoxicity (3-BP, DCA), nephrotoxicity (3-BP), cardiotoxicity (2DG) and systemic metabolic disturbances such as hypoglycemia (3-BP, 2DG) [151,190,191]. In addition to direct toxicity, inhibition of glycolysis may induce compensatory metabolic adaptations in normal tissues (e.g., increased oxidative phosphorylation or alternative substrate utilization), which are often insufficient in high-energy-demand organs and further contribute to a narrow therapeutic window [192]. These adverse effects limit the translation of these inhibitors to the clinical setting and require special administration or combination with other agents, both in order to decrease the administered dose.

A notable candidate currently undergoing clinical evaluation is AZD3965, an MCT1 inhibitor, which has already entered early-phase clinical trials. Its preclinical activity in ATC models, where it inhibits proliferation, together with a relatively targeted mechanism focused on lactate export rather than global glycolysis inhibition, makes it particularly attractive for clinical translation. Importantly, its mechanism is thought to preferentially affect highly glycolytic tumor cells while partially sparing normal tissues, improving its therapeutic index compared with upstream glycolytic blockers [165,166].

Another investigated compound is acriflavine, which has demonstrated antiproliferative activity in preliminary preclinical models, though its in vivo evaluation remains limited. Its mechanism of action primarily involves the disruption of HIF-1α dimerization, which downregulates downstream glycolytic targets, with an additional suppression of MCT4 expression. Consequently, this intervention may impair both the upstream regulation of the WE and downstream lactate export [168].

2DG demonstrated consistent anti-tumor effects and synergy with radiotherapy in in vitro models [193]. However, its clinical potential is limited by systemic toxicity and metabolic intolerance due to its direct interference with fundamental glucose metabolism, which reduces its therapeutic window despite repeated preclinical validation [190].

Other inhibitors, such as or 3-BP or BAY-876, although present with good preclinical results, are limited mainly by increased toxicity [191,194]. Additionally, natural agents such as shikonin or apigenin suffer from limited pharmacokinetic optimization, reducing their suitability for clinical development [195,196].

In contrast, compounds like alisertib, an Aurora A inhibitor, benefit from prior clinical oncology experience, as it was used for neuroblastoma, small cell lung cancer, prostate cancer, and breast cancer among others [197]. Alisertib may be more suitable for repurposing, especially in combination strategies that indirectly affect tumor metabolism [198].

Overall, current evidence suggests that viable therapeutic strategies rely on agents that selectively disrupt tumor-specific metabolic dependencies while maintaining acceptable systemic tolerability. Furthermore, molecules with established clinical safety profiles present significant utility, particularly when integrated into combination regimens with conventional standard-of-care therapies.

### 6.3. Future Treatment Directions

While significant efforts are focused on selecting inhibitors with the most favorable safety profiles, systemic toxicity remains a persistent challenge in current treatment models. Modern research is pivoted toward developing safer therapeutic formulations that either mitigate these widespread side effects or target the tumor directly, bypassing the need for systemic administration.

A possible solution to this problem could be vehicle-targeted therapy or intratumoral administration, such as brachytherapy, which would limit systemic effects, decrease the risk of complications, and improve patient compliance, as well as administration of lower doses in combination with classical therapies such as radio or immunotherapy, although the rapid progression of ATC and PDTC limits such approaches.

Innovative drug delivery methods are currently being engineered to bypass the systemic toxicity associated with potent antineoplastic agents [199]. By leveraging the biochemical composition of the tumor microenvironment, lactate-sensitive gold/mesoporous Janus nanoparticles are attracted to the tumor site due to the high intratumoral lactate concentration [200], while magnetic liposomes could be directed towards the tumor using strong magnets placed on the neck of patients [201]. Cationic nanocarriers exploit the negative charge present on cancer cells, thus delivering the inhibitors [202]. These precision targeting strategies open new possibilities for the treatment of aggressive cancers, which require aggressive therapies.

Combining brachytherapy with localized drug delivery is one future direction that has attractive advantages. Brachytherapy is an already established procedure with proven benefits by concentrating radiation exposure to the tumor tissue, while minimizing healthy tissue exposure by using implantable “seeds” [203].

Creating brachytherapy seeds that combine the radioisotopes with drugs that ellute overtime would minimize the impact of cancer therapy on patients by reducing the number of visits (one implantation that handles both the radiation therapy and chemotherapy, as opposed to a visit for radiotherapy and multiple visits for chemotherapy administration), would reduce the systemic effects of the chemotherapy by minimizing systemic concentrations, and would improve the pharmacodynamic profile of the drugs, by ensuring higher doses directed in the target tissue [204]. A similar technique could be expanded to include WE inhibitors in the formulation.

By avoiding systemic administration, these options not only require lower drug doses while maximizing intratumor concentrations but also target cancer cells specifically, thereby offering promising therapeutic potential for ATC.

However, the clinical utility of this approach is limited by the rapid development of distant metastases and extensive local invasion, which often render purely locoregional therapies insufficient. Combination therapies are conceptually attractive, particularly given preclinical evidence that WE inhibition may enhance tumor immunogenicity and increase radiosensitivity. Overall, the synergy is mainly driven by metabolic reprogramming of the TME, making cancer cells less adaptable and more vulnerable to standard therapies. However, clinical translation remains limited, as overlapping toxicities between combined modalities, together with the reliance of activated immune cells on glycolytic metabolism, may complicate therapeutic efficacy and safety [205,206].

**Figure 3:**
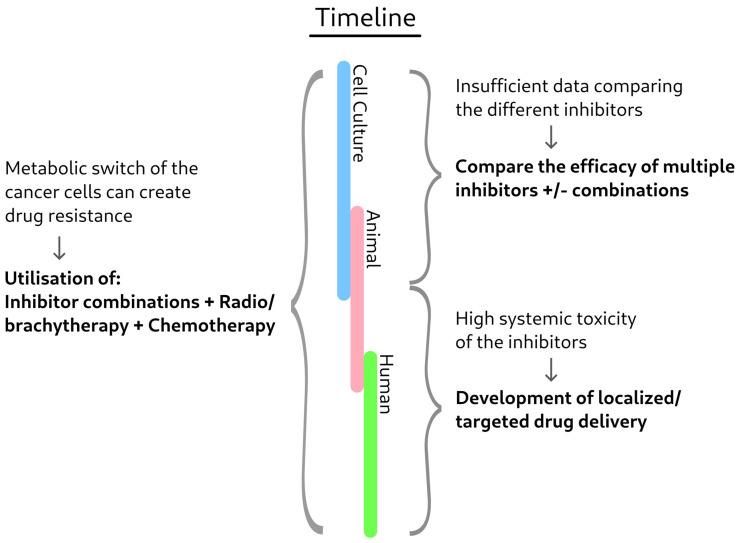
Summary of the current challenges of using WE inhibitors in the treatment of ATC and suggested approaches (in bold) to solve them, in a tentative timeline with adequate experimental designs to answer the research questions. Nota bene: The length of the timeline bars does not correlate with actual duration, but rather indicates the logical, sequential order of experiments across different translational phases (Cell Culture, Animal models, and Human application).

In Figure 3 we have summarized the current limitations and challenges in ATC treatment, as well as possible strategies for overcoming them.

Another promising research direction in ATC is the targeting of upstream oncogenic drivers that, in addition to promoting tumor growth and progression, also contribute to metabolic reprogramming. The molecular heterogeneity of ATC and PDTC suggests that future metabolic therapies may benefit from a genotype-based approach, either through direct inhibition of oncogenic drivers or through combinations of targeted therapies with inhibitors of glycolytic metabolism. For example, tumors harboring BRAFV600E mutations may be particularly susceptible to combinations of BRAF/MEK inhibitors, such as dabrafenib/trametinib, and glycolysis-targeting agents, as BRAFV600E signaling has been shown to promote aerobic glycolysis through upregulation of glycolytic enzymes and glucose utilization [22,45]. Similarly, RAS-mutated tumors, which frequently activate downstream PI3K/AKT/mTOR signaling, may benefit from combined targeting of oncogenic signaling and metabolic pathways. Inhibitors such as capivasertib, an AKT inhibitor that has shown clinical activity in selected solid tumors, may represent a potential therapeutic strategy in combination with agents targeting glycolytic metabolism in ATC [46].

TP53-deficient tumors may display increased dependence on glycolytic metabolism and heightened sensitivity to therapies that exploit metabolic stress, as loss of p53 function shifts cellular energy production toward glycolysis and reduces metabolic flexibility [80,207].

Tumors characterized by elevated HIF-1α activity may be particularly vulnerable to strategies targeting hypoxia-driven metabolic adaptation, glucose transport, or lactate metabolism [40,41]. Acriflavine has already been discussed above in this regard.

Future studies should aim to identify genotype-specific metabolic dependencies and determine whether molecular stratification can improve the efficacy of therapies targeting WE in aggressive thyroid cancers.

These challenges underscore the need to define optimal therapeutic windows and to further validate such approaches in well-designed clinical trials. Given the rarity and aggressiveness of ATC, future studies should prioritize carefully stratified patient cohorts, ideally focusing on non-metastatic or minimally disseminated disease where locoregional control remains achievable. Randomized study designs, although challenging in this context due to limited patient numbers, should aim to evaluate clinically meaningful endpoints such as objective tumor response, reduction in tumor burden enabling surgical intervention, progression-free survival, and overall survival, including long-term follow-up where feasible.

Despite significant advancements in oncological treatments, establishing optimal therapeutic protocols for aggressive malignancies such as ATC remains a dire challenge. More research is needed to assess the therapeutic potential of novel inhibitors, especially in combination with classical therapies.

## Figures and Tables

**Table 1:** Synthesis of main mutations and their comparative frequency in ATC and PDTC, vs. differentiated thyroid neoplasms.

Mutation	Differentiated Thyroid Neoplasms	PDTC	ATC	References
BRAF^V600E^	60–80% PTC	≈33%	≈45%	[16,19,20,47]
RAS	Common in FTC, benign or malignant lesions	24–27%	24–27%	[16,19,20,47]
PAX8/PPARγ	20–40% in FTC	<5%	<5%	[48,49]
TERT	Rare 11% PTC	≈40%	65–73%	[29,47,50]
TP53	Rare	≈16%	48–65%	[36,47]
ATM	Rare	<10%	<10%	[16,19,20]
PI3K/AKT/mTOR	Rare	5–15%	10–17%	[16,51,52,53]
PTEN	Rare	<10%	Rare	[51,52,53]

ATC, Anaplastic Thyroid Cancer; FTC, follicular thyroid carcinoma; PTC, papillary thyroid carcinoma; PDTC, poorly differentiated thyroid cancer.

**Table 2:** Summary of the central metabolic nodes involved in WE in ATC/PDTC, representative inhibitors targeting these pathways, and their reported preclinical antitumor effects.

Metabolic Target	Role in Warburg Effect	Main Antitumor Effects of Specific Inhibitors (Preclinical)	Therapeutic Relevance (Preclinical Evidence) Cancer	References
GLUT1/GLUT3	Increased glucose uptake	Reduced glucose uptake, growth inhibition	Inhibitors could serve as potential radio/chemosensitizer	[102,103,104]
HK2	Initiates glycolysis; couples to mitochondria via VDAC1	ATP depletion, apoptosis, reduced proliferation, impaired migration/invasion	Especially relevant in aggressive, hypoxic, glycolysis-addicted tumors Inhibitors could serve as potential radio/chemosensitizer	[105,106]
PKM2	Central hub of metabolic plasticity and biosynthesis in aggressive tumors, rate limiting enzyme	Inhibits PKM2 activity, reduces glucose uptake/lactate/ATP, induces apoptosis, suppresses tumor growth	Regulates metabolism and biosynthesis Inhibitors could serve as potential radio/chemosensitizer	[107,108]
PFKFB3	Glycolytic flux regulation, indirectly activating PFK1	Reduced proliferation, metabolic stress	Links oncogenic signaling to metabolism Induces proliferation, migration, and glycolytic activation	[109,110]
PDK1	Inhibits PDC blocking mitochondrial entry of pyruvate; redirects flux to lactate.	Reactivates mitochondria, increases ROS production, triggers apoptosis.	Reverses the glycolytic phenotype Inhibitors could serve as potential radio/chemosensitizer	[111,112]
LDHA	Lactate production, NAD^+^ regeneration	Reduces lactate, impairs tumor growth, alters redox balance, enhances antitumor immunity	Impacts acidic, immunosuppressive microenvironment and invasiveness	[113,114,115]
MCT1/MCT4	Mediate lactate export/import	Intracellular acidification, metabolic crisis; disrupts lactate shuttling; influences metastasis	Inhibitors could be combined with glycolysis inhibitors or with immunotherapy	[116,117]
HIF-1α (indirect effect)	Hypoxia-driven metabolic rewiring Upregulation of GLUT1, HK2, PFKFB3, LDHA, MCTs Treatment resistance (chemio and radiotherapy)	Decreases expression of glycolytic genes, reduces glycolysis, growth and angiogenesis	Central driver of hypoxic, glycolytic phenotype Attractive upstream target Inhibitors could serve as potential radio/chemosensitizer	[41,118]

2-DG, 2-Deoxy-D-glucose; 3-BP, 3-Bromopyruvate; 3PO, 3-(3-pyridinyl)-1-(4-pyridinyl)-2-propen-1-one; ATC, Anaplastic Thyroid Cancer; ATP, Adenosine triphosphate; GLUT1/3, Glucose transporters 1 and 3; HIF-1α, Hypoxia-inducible factor 1-alpha; HK2, Hexokinase 2; LDHA, Lactate dehydrogenase A; MCT1/4, Monocarboxylate transporters 1 and 4; NAD^+^, Nicotinamide adenine dinucleotide (oxidized); PDC, Pyruvate dehydro-genase complex; PDK1, Pyruvate dehydrogenase kinase 1; PFK1, Phosphofructokinase 1; PFKFB3, 6-phosphofructo-2-kinase/fructose-2,6-biphosphatase 3; PKM2, Pyruvate kinase M2; PDTC, Poorly differentiated thyroid cancer; ROS, Reactive ox-ygen species; VDAC1, Voltage-dependent anion channel 1.

**Table 3:** Summary of the descriptive studies investigating the expression levels of key glycolytic enzymes associated with the WE in ATC and PDTC (cell lines and patient samples).

Name of Study	Enzyme/Molecule Studied	Cell Line/Tissue Used	Details	Quantification Method	Conclusion	Reference
Glycolysis-related protein expression in thyroid cancer	GLUT	Thyroid malignancies biopsies	556 patients 19 ATC 23 PDTC	Immunohistochemical staining	Highest staining levels in ATC patients, followed by PDTC	[104]
	HK2					
	MCT4					
Expression of the GLUT1 glucose transporter, p63 and p53 in thyroid carcinomas	GLUT1	Thyroid malignancies biopsies	86 patients 7 ATC 9 PDTC	Immunohistochemical staining	Highest staining levels in ATC patients, followed by PDTC	[122]
Expression analysis of facilitative glucose transporters (GLUTs) in human thyroid carcinoma cell lines and primary tumors	GLUT1/3	Thyroid tumor cell lines	ARO and FRO (ATC), NPA (PSTC), WRO (FTC), and TT (medullary thyroid cancer)	RT-PCR (GLUT mRNA), Western blot	Significant increase in GLUT1, slight increase in GLUT3 mRNA in cancer samples, with higher values in ATC than in PTC or FTC	[123]
		Thyroid malignancies or bening biopsies	157 patients 6 ATC			
Hexokinase isoenzymes from anaplastic and differentiated medullary thyroid carcinoma in the rat.	HK1/2	Rat ATC and medullary carcinoma		Fast protein liquid chromatography	No major difference in kinetic properties of the enzyme between the tumors Increased HK2 in ATC	[130]
Glucose Increases Hexokinase-2 Expression and Glycolytic Capacity in Anaplastic Thyroid Cancer	HK2	ATC cell lines	JL30 and 8505C in high (25 mM) or low (3 mM) glucose	Fluorescent in situ hybridization	Higher HK2 expression in high glucose medium	[132]
RBX1 regulates PKM alternative splicing to facilitate anaplastic thyroid carcinoma metastasis and aerobic glycolysis by destroying the SMAR1/HDAC6 complex	PKM2 RBX1	ATC cell lines	C643, CAL62, 8505C KMH2, KMH-5M, and BHT101	Western blotting, Immunohistochemistry shRNA Functional tests	RBX1 promotes ATC development by increasing the WE RBX1 promotes ATC development by increasing the WE	[137]
		Thyroid malignancies or bening biopsies	30 ATC patient samples			
Phosphorylation of pyruvate kinase M2 and lactate dehydrogenase A by fibroblast growth factor receptor 1 in benign and malignant thyroid tissue	PKM2	Thyroid malignancies biopsies	77 patients 16 ATC	Western Blot SYBR Green RT-PCR	Highest levels found in ATC	[142]
	LDH				Highest levels found in ATC	
Mitochondrial Metabolism as a Treatment Target in Anaplastic Thyroid Cancer	MCT	Thyroid malignancies biopsies	35 patients 15 ATC	Immunohistochemistry staining	Highest levels found in ATC, for both human and xenograft tissue	[126]
		ATC mouse orthotopic xenografts				
PFKFB3 facilitates cell proliferation and migration in anaplastic thyroid carcinoma via the WNT/β-catenin signaling pathway	PFKFB3	ATC mouse orthotopic xenografts		Western Blot Immunohistochemistry Staining Luciferase reporter	High levels found in ATC PFKFB3 stimulates ATC cells proliferation and migration	[135]
		Cell cultures	CAL62, KMH-5M			

ATC, Anaplastic Thyroid Cancer; FTC, Follicular thyroid carcinoma; GLUT, Glucose transporter; HK1/2, Hexokinase 1/2; LDH, Lactate dehydro-genase; MCT, Monocarboxylate transporter; MCT4, Monocarboxylate transporter 4; mRNA, Messenger ribonucleic acid; PDTC, Poorly differentiated thyroid cancer; PFKFB3, 6-phosphofructo-2-kinase/fructose-2,6-biphosphatase 3; PKM2, Pyruvate kinase M2; PTC, Papillary thyroid carcinoma; RBX1, Ring-box protein 1; RT-PCR, Reverse transcription polymerase chain reaction; shRNA, Short hairpin RNA; WE, Warburg effect.

**Table 4:** Summary of direct and indirect Warburg effect inhibitors in ATC, their mechanism of action, preclinical experimental models used in the literature, and available toxicity data The Warburg effect inhibitors have been grouped into synthetic and natural compounds. Compounds that exert an indirect inhibitory effect on glycolysis have been marked with an asterisk (*).

Paper	WE Inhibitor	Mechanism of Action	Experimental Model	Reference
Natural Compounds				
Shikonin Inhibits the Growth of Anaplastic Thyroid Carcinoma Cells by Promoting Ferroptosis and Inhibiting Glycolysis	Shikonin	Inhibits aerobic glycolysis in ATC cells by reducing expression of - PKM2 in both cell lines in a dose-dependent manner - GLUT1 in CAL62 cells at 3 and 5 μM - GLUT1 8505C cells at all concentrations Leads to decreased glucose uptake and lactate production. Increases ROS production	CAL62 and 8505C human ATC cell line—SKN (1, 3, and 5 μM) for 24 h.	[159]
		Significantly slows tumor growth rate Lowers GPX4, TXNRD1, NF-κb, PKM2, and GLUT1 expression	Female Balb/c mice (4–5 W) treated with 5 × 10^6^ CAL62 cells	
The Effect of Apigenin on Glycometabolism and Cell Death in an Anaplastic Thyroid Cancer Cell Line	Apigenin *	Inhibits proliferation and migration Decreases glucose uptake Downregulates GLUT1 and GLUT3 expression	SW1736 ATC cells	[160]
Study on the Antitumor Effect and Glycolysis of Andrographolide in Anaplastic Thyroid Carcinoma	Andrographolide *	HK2, PKM2, PFKM, and LDHA expression reduction G2/M cell cycle arrest Caspase-3/PARP pathway apoptosis induction	ATC cell lines 8505C and CAL62	[188]
Synthetic Compounds				
Glycolytic Inhibition with 3-Bromopyruvate Suppresses Tumor Growth and Improves Survival in a Murine Model of Anaplastic Thyroid Cancer	3BP	Reduces growth by 90% at both glucose concentrations in all tested ATC and PDTC cells	In vitro: ATC cell line 8505C PDTC cell line BCPAP JL30 cells	[174]
	3BP combined with a ketogenic diet	Inhibits tumor growth and prolongs survival	In vivo: Orthotopic xenograft model in Female J:NU athymic nude mice (6 weeks old) using 8505C cells	
Glycolytic Inhibition Alters Anaplastic Thyroid Carcinoma Tumor Metabolism and Improves Response to Conventional Chemotherapy and Radiation	2-DG	2-DG acts as a competitive inhibitor of glucose, thus blocking the glycolytic pathway. 2-DG sensitized cells to radiotherapy (in vitro)	8 ATC cell Lines with BRAF V600E Mutations in 4 cell lines (U-HTH83, U-HTH104, 8505C, And SW1726) and identified nonsynonymous mutations In KIT (D816H; U-HTH7) and N-RAS (Q61R; U-HTH7)	[172]
		Significant decrease in ATC xenograft Reducing potential by 30% as shown by decreased HP-MRI Signal for less than 24 h	Male athymic nude mice (8–12 weeks) injected with U-HTH83 luciferase expressing cells (xenograft model)	
Targeting Tumor Hypoxia Inhibits Aggressive Phenotype of Dedifferentiated Thyroid Cancer	Acriflavine	Inhibits HIF-1α signaling pathways, leading to downregulation of genes involved in glucose metabolism and cell proliferation Inhibits MCT4	ATC cell lines: CAL62 and 8305C PTC cell lines: TPC-1 and K1 normal thyroid Cell-Nthy-ori 3-1 (control)	[168]
		Reduces tumor volume (CAL62)	Female athymic BALB/c nude mice (n = 14) with an age of 4 to 5 weeks injected with 0.2 mL of cell suspension containing 5 × 10^6^ CAL62 cells	
PFKFB3 Facilitates Cell Proliferation and Migration in Anaplastic Thyroid Carcinoma via the WNT/Β-catenin Signaling Pathway	KAN0438757 *	Decreases PFKFB3 protein expression and reduces lactate production, Disrupts DNA repair by affecting RNA reductase M2 and dntp Inhibits ATC cell proliferation and migration Inhibits WNT/β-catenin signaling	ATC cell lines KHM-5M and CAL62	[135]
		Suppresses ATC tumor growth Downregulate p-AKT, FN1, MMP9 and Cyclind1 Decreased lactate production Suppressed the expression of p-AKT (ser473) GSK3α/β, nuclear β-catenin, FN1, MMP9 and cyclin D1 Reduces the translocation of β-catenin into the nucleus	Six-week-old female nude mice (n = 6) via the subcutaneous route (1 × 10^6^ Cal-62 cells in 100 μL PBS)	
RBX1 Regulates PKM Alternative Splicing to Facilitate Anaplastic Thyroid Carcinoma Metastasis and Aerobic Glycolysis by Destroying the SMAR1/HDAC6 Complex	2-DG	2-DG acts as a competitive inhibitor of glucose thus blocking the glycolytic pathway.	8305C/HA-RBX1 cells CAL62/shrbx1 cells	[137]
	Proteasome inhibitor MG132 *	Silences RBX1, which inhibits SMAR1 degradation	8305C cells + P-RBX1 CAL62 cells transfected with HA-RBX1 or shrbx1 plasmids	
Disrupting Glycolysis and DNA Repair in Anaplastic Thyroid Cancer with Nucleus-Targeting Platinum Nanoclusters	Ph-responsive and nucleus-targeting platinum nanocluster (Pt@TAT/speg) *	Reduces intracellular LDH activity Reduces lactate and ATP production	ATC cell line 8505C	[186]
		Reduces tumor intensity Reduces tumor growth	BALB/c athymic nude mice (female, 5–6 weeks) injected with 5 × 10^5^ luciferase-expressing 8505C cells to establish the orthotopic xenograft	
Autophagy Sustains Mitochondrial Respiration and Determines Resistance to BRAFV600E Inhibition in Thyroid Carcinoma Cells	UK5099	Inhibits MPC Impairs glucose usage Reduces oxygen consumption rate	ATC cell line BHT101	[173]
	Etomoxir + 2-DG	Decreases cell viability 2DG competitively inhibits glucose consumption Etomoxir inhibits CPT1 (carnitine palmitoyltransferase 1), avoiding transport of long chain FA to the mitochondria for their oxidation		
	2-DG	Decreases cell viability Competitively inhibits glucose consumption		
	2-DG	Greater viability decrease than BHT101 cells Competitively inhibits glucose consumption	ATC cell line BHT101 with ATG7-KO	
	Vemurafenib/PLX4032 *	Inhibits glycolysis Reduces lactate production Reduces cell viability More effective in ATG7-KO cells	ATC cell line BHT101 ATC cell line BHT101 with ATG7-KO	
Mannose Enhances Anti-Tumor Effect of PLX4032 in Anaplastic Thyroid Cancer	Vemurafenib/PLX4032 *	Mannose increased PLX4032-induced apoptosis, lowered cell migration PLX4032 enhanced mannose-induced glycolysis suppression PLX4032 inhibits PMI activity	ATC cells 8305C and 8505C	[175]
	PLX4032 + mannose *	Reduced tumor size and proliferation rate to a greater degree than PLX4032 alone	Female BALB/c/nu nude mice, aged 4–5 weeks treated with s.c. 200 μL of 5 × 10^6^ 8305C or 8505C cells	
Targeting Metabolism by B-Raf Inhibitors and Diclofenac Restrains the Viability of BRAF-Mutated Thyroid Carcinomas with Hif-1α-Mediated Glycolytic Phenotype	PLX4032/vemurafenib	Reduces cell viability Impairs the glycolytic phenotype Transcriptionally represses glycolysis-related genes reduces glucose uptake Reduces lactate efflux	ATC cell line 8505C	[170]
	Trametinib *			
	Diclofenac *	Impairs glucose uptake Impairs lactate excretion		
Effect of Lactate Export Inhibition on Anaplastic Thyroid Cancer Growth and Metabolism	AZD3965	Inhibition of MCT1 Reduced proliferation in all tested lines	BRAF V600E mutated ATC cell lines 8505C, JL30, and TCO1	[167]
	Acriflavine	Inhibition of MCT4 Reduced cell proliferation (8505C, JL30 cells) Reduced lactate levels Suppresses glycolysis		
	Syrosingopine	Inhibition of MCT1/4 Reduced cell proliferation (8505C and TCO1 cells)		
Development of a Rational Strategy for Integration of Lactate Dehydrogenase A Suppression into Therapeutic Algorithms for Head and Neck Cancer	Doxycycline-inducible LDHA knockdown shRNA system	Partially suppresses LDH activity Inhibits cell proliferation Increases radiation effectiveness	ATC cell line Hth83 cells	[171]
RON Receptor Tyrosine Kinase Regulates Glycolysis through MAPK/CREB Signaling to Affect Ferroptosis and Chemotherapy Sensitivity of Thyroid Cancer Cells	RON-specific siRNA *	Reduced extracellular acidification rate, lactic acid and glucose levels Downregulated HK2 and PKM2 expression Lowered ferroptosis by inhibiting HK2 and downregulating MAPK/CREB signaling Elevated cell sensitivity to chemotherapy	8305C ACT cell line	[187]
Targeting Aurora-A Inhibits Tumor Progression and Sensitizes Thyroid Carcinoma to Sorafenib by Decreasing PFKFB3-Mediated Glycolysis	Alisertib *	Aurora kinase inhibitor Inhibits proliferation, invasion, migration	Anaplastic Thyroid Cancer cell lines (8305C, 8505C and CAL62)	[163]
		Inhibited tumor proliferation	Tumor xenograft model, NSG female mice (4 weeks old) were injected subcutaneously at 100 μL PBS with 1 × 10^5^ CAL62 or 8305C cells	
Glucose Transporter 1 Inhibitors Induce Autophagy and Synergize With Lenvatinib in Thyroid Cancer Cells	BAY-876	Inhibits GLUT1 G2/M cell cycle arrest Autophagy activation Synergy with lenvatinib reduces ATC cell migration	ATC cell line 8505C Papillary thyroid cancer cell lines TPC-1, B-CPAP Spheroid formation in extracellular matrix (Cultrex) for 3D invasion assays	[158]
	STF-31			
circCCDC66 Promotes Thyroid Cancer Cell Proliferation, Migratory and Invasive Abilities and Glycolysis through the miR-211-5p/PDK4 Axis	Si-circccdc66	circccdc66 knockdown inhibits TC glycolysis circccdc66 serves as a sponge of mir-211-5p to promote PDK4 expression	ATC cell line CAL62	[183]

2-DG, 2-Deoxy-D-glucose; 3-BP, 3-bromopyruvate; ATC, Anaplastic Thyroid Cancer; ATG7-KO, Autophagy-related 7 knockout; ATP, Adenosine triphosphate; circCCDC66, Circular RNA CCDC66; CPT1, Carnitine palmitoyltransferase 1; DMSO, Dimethyl sulfoxide; FN1, Fibronectin 1; GLUT1/3, Glucose transporters 1 and 3; GPX4, Glutathione peroxidase 4; GSK3α/β, Glycogen synthase kinase 3 alpha/beta; HDAC6, Histone deacetylase 6; HIF-1α, Hypoxia-inducible factor 1-alpha; HK2, Hexokinase 2; HP-MRI, Hyperpolarized Magnetic Resonance Imaging; IC_50_, Half maximal inhibitory concentration; LDH, Lactate dehydrogenase; LDHA, Lactate dehydrogenase A; MAPK/CREB, Mitogen-activated protein kinase/cAMP response element-binding protein; MCT4, Monocarboxylate transporter 4; MMP9, Matrix metalloproteinase 9; MPC, Mitochondrial pyruvate carrier; NF-κB, Nuclear factor kappa-light-chain-enhancer of activated B cells; p-AKT, Phosphorylated protein kinase B; PARP, Poly (ADP-ribose) polymerase; PDK4, Pyruvate dehydrogenase kinase 4. PDTC, Poorly differentiated thyroid cancer; PFKFB3, 6-phosphofructo-2-kinase/fructose-2,6-biphosphatase 3; PFKM, Phosphofructokinase, muscle type; PKM2, Pyruvate kinase M2; PMI, Phosphomannose isomerase; RBX1, Ring-box protein 1; RON, Recepteur d’Origine Nantais (Receptor tyrosine kinase); ROS, Reactive oxygen species; shRNA, short hairpin RNA; siRNA, Small interfering RNA; SMAR1, Scaffold/matrix attachment region-binding protein 1; TXNRD1, Thioredoxin reductase 1; WNT/β-catenin, Wingless-related integration site/beta-catenin.

## Data Availability

No new data were created or analyzed in this study. Data sharing is not applicable to this article.

## Abbreviations

The following abbreviations are used in this manuscript:

2-DG	2-Deoxyglucose
3-PO	3-(3-pyridinyl)-1-(4-pyridinyl)-2-propen-1-one
3-BP	3-Bromopyruvate
PFKFB3	6-phosphofructo-2-kinase/fructose-2,6-biphosphatase 3
ADP	Adenosine Diphosphate
ATP	Adenosine Triphosphate
ATC	Anaplastic thyroid carcinoma
ATG7-KO	Autophagy-related 7 knockout
CAF	cancer-associated fibroblast
CPT1,	Carnitine palmitoyltransferase 1
circCCDC66	Circular RNA CCDC66
CD4	Cluster of differentiation 4, Helper T cells
CD8	Cluster of differentiation 8, Cytotoxic T cells
DCA	Dichloroacetate
DMSO	Dimethyl sulfoxide
EMT	Epithelial–mesenchymal transition
FN1	Fibronectin 1
FTC	Follicular thyroid cancer
F-1,6BP	Fructose-1,6-Biphosphate
F-2,6BP	Fructose-2,6-Biphosphate
Fructose-6P	Fructose-6-Phosphate
GLUT	Glucose transporter
Glucose-6P	Glucose-6-Phosphate
GPX4	Glutathione peroxidase 4
GSK3α/β	Glycogen synthase kinase 3 alpha/beta
IC50	Half maximal inhibitory concentration
HK	Hexokinase
HDAC6	Histone deacetylase 6
HP-MRI	Hyperpolarized Magnetic Resonance Imaging
HIF-1α	Hypoxia Inducible Factor 1α
IL	Interleukin
LDH	Lactate dehydrogenase
LDHA	Lactate dehydrogenase A
LDHB	Lactate dehydrogenase B
MMP	Matrix metalloproteinase
MMP9	Matrix metalloproteinase 9
mRNA	Messenger ribonucleic acid
MAPK/CREB	Mitogen-activated protein kinase/cAMP response element-binding protein
MCT	Monocarboxylate transporter
MCT1	Monocarboxylate transporter 1
MCT4	Monocarboxylate transporter 4
MPC	MPC, Mitochondrial pyruvate carrier
MDSC	Myeloid-derived suppressor cell
NK	Natural killer cells
NAD^+^	Nicotinamide adenine dinucleotide (oxidized)
NF-κB	Nuclear factor kappa-light-chain-enhancer of activated B cells
PTC	Papillary thyroid cancer
PDK4	PDK4, Pyruvate dehydrogenase kinase 4.
PPP	Pentose phosphate pathway
PEP	Phosphoenolpyruvate
PFK1	Phosphofructokinase 1
PFKM	Phosphofructokinase, muscle type
PFK1	Phosphofructokinase-1
PHI	Phosphohexose Isomerase
PMI	Phosphomannose isomerase
p-AKT	Phosphorylated protein kinase B
Pt@TAT/sPEG	Ph-responsive and nucleus-targeting platinum nanocluster
PDGF	Platelet-derived growth factor
PARP	Poly (ADP-ribose) polymerase
PDCT	Poorly differentiated thyroid carcinoma
PDH	Pyruvate Dehydrogenase
PDC	Pyruvate dehydrogenase complex
PDK1	Pyruvate dehydrogenase kinase 1
PKM	Pyruvate kinase M
ROS	Reactive oxygen species
RON	Recepteur d’Origine Nantais (Receptor tyrosine kinase)
Treg	Regulatory T cell
RT-PCR	Reverse transcription polymerase chain reaction
RBX1	Ring-box protein 1
SMAR1	Scaffold/matrix attachment region-binding protein 1
shRNA	Short hairpin RNA
siRNA	Small interfering RNA
TXNRD1	Thioredoxin reductase 1
TGF-β	Transforming growth factor β
TCA Cycle	Tricarboxylic acid cycle
TSHR	TSH receptor
TME	Tumor microenvironment
VEGF	Vascular endothelial growth factor
VDAC1	Voltage-dependent anion channel 1
WE	Warburg effect
WNT/β-catenin	Wingless-related integration site/beta-catenin
